# The Genetic Polymorphisms in the *MIR17HG* Gene Are Associated with the Risk of Head and Neck Squamous Cell Carcinoma in the Chinese Han Population

**DOI:** 10.1155/2020/2329196

**Published:** 2020-11-24

**Authors:** Chongwen Xu, Peng Han, Wanli Ren, Hao Dai, Yanxia Bai, Zhen Shen, Baiya Li, Yuan Shao

**Affiliations:** Department of Otolaryngology Head and Neck Surgery, The First Affiliated Hospital, Xi'an Jiaotong University, Xi'an, 710061 Shaanxi, China

## Abstract

**Purpose:**

Head and neck squamous cell carcinoma (HNSCC) is the most common malignant tumors in the world. Genetic variants have an important role in HNSCC progression. Our study is aimed at exploring the relationship between *MIR17HG* polymorphisms and HNSCC risk in the Chinese Han population.

**Methods:**

We recruited 537 HNSCC cases and 533 healthy subjects to detect the correlation of six polymorphisms in *MIR17HG* with HNSCC susceptibility. The associations were evaluated by computing odds ratios (ORs) and 95% confidence intervals (CIs) using logistic regression analysis.

**Results:**

Our study revealed that rs7336610 (OR 1.77, 95%CI = 1.09‐2.86, and *p* = 0.021) and rs1428 (OR 1.73, 95%CI = 1.07‐2.81, and *p* = 0.025) are strongly associated with increased susceptibility to HNSCC in men. Besides, rs17735387 played a crucial protective role in stage III/IV HNSCC patients (OR 0.34, 95%CI = 0.12‐0.95, and *p* = 0.040) compared with stage I/II.

**Conclusion:**

Our study firstly indicated that *MIR17HG* polymorphisms are significantly associated with HNSCC susceptibility, which suggests that *MIR17HG* has a potential role in the occurrence of HNSCC.

## 1. Introduction

Head and neck squamous cell carcinoma (HNSCC) is the most common malignant tumors, which includes tumors from the oral cavity, hypopharynx, pharynx, and larynx. According to epidemiological statistics, HNSCC ranks sixth among malignant tumors in the world [1]. In China, nasopharyngeal carcinoma, laryngeal carcinoma, oral cancer, and thyroid cancer are common HNSCC. Most patients with HNSCC were diagnosed at middle and advanced stages, resulting in markedly high morbidity and mortality [2]. HNSCC patients usually have typical risk factors, such as smoking, drinking, and or human papillomavirus (HPV) infection [3, 4]. However, people without known risk factors also developed HNSCC. And increased studies revealed that genetic factors have a crucial effect on the occurrence of HNSCC. Long noncoding RNA (lncRNA) is a noncoding RNA longer than 200 nucleotides, which can regulate the occurrence and development of human cancers. Now, some studies indicated that lncRNAs participate in the progression and development of HNSCC by regulating the behavior of HNSCC cells [5–7]. Besides, numerous studies suggested that polymorphisms of some lncRNAs included in *PTENP1*, and *HOTAIR* are significantly associated with HNSCC susceptibility [8, 9]. Taken together, it suggests that genetic variants of lncRNAs may have a crucial role in HNSCC progression.

miR-17-92a-1 cluster host gene (MIR17HG), located on the 13q31.3 of the human chromosome, is a member of lncRNAs that contributes to the occurrence of many human tumors including colorectal cancer, breast cancer, and multiple myeloma through regulating cell survival, differentiation, and proliferation [10–12]. Besides, the human gene database showed that *MIR17HG* was significantly expressed in the head and neck (https://www.genecards.org/cgibin/carddisp.pl?gene=MIR17HG&keywords=MIR17HG). A recent study indicated that MIR17HG could affect the abnormal expression of miR-17-92 gene cluster miR-17 and therefore contributes to the development of human tumors [13]. Wang et al. found that the silencing of miR-17 can promote cell apoptosis and inhibit cell proliferation in laryngeal squamous cell carcinoma [14]. MIR17HG acted as a tumor suppressor lncRNA in HPV-positive HNSCC tumors compared to HPV-negative tumors, which plays a distinct role in HPV-related HNSCC [15]. Genetic variants within genes can affect the expression or structure of the genes, which may result in the progression of cancers. Moreover, previous studies have revealed that the polymorphisms of *MIR17HG* are markedly related to the occurrence of cancers [13, 16, 17]. Taken together, we speculate that the *MIR17HG* genetic variant may have a potential role in the HNSCC progression. To our knowledge, there is no study on the association between the *MIR17HG* polymorphisms and HNSCC susceptibility.

To better know the effect of *MIR17HG* genetic variant on the risk of HNSCC in the Chinese population. In this case-control study, we selected six (single nucleotide polymorphisms) SNPs (rs75267932, rs17735387, rs7336610, rs72640334, rs7318578, and rs1428) in *MIR17HG* from 1000 Genomes Project with minor allele frequencies > 5%, *r*^2^ < 0.8, and Hardy − Weinberg equilibrium > 0.05. MassARRAY platform was performed to detect the SNP genotyping. We then studied the association of *MIR17HG* SNPs with the susceptibility of HNSCC. Finally, we evaluated the relationship of *MIR17HG* variants with the risk of HNSCC stratified by age, gender, and pathological grade. Our present work will give new scientific evidence for the molecular mechanism of HNSCC development in the Chinese population.

## 2. Materials and Methods

### 2.1. Study Population

A total of 1070 participants included in 537 unrelated Chinese HNSCC patients (43 laryngeal SCC, 77 nasopharyngeal SCC, 398 thyroid SCC, and 19 parotid SCC) and 533 age-sex matched healthy controls were recruited from the First Affiliated Hospital of Xi'an Jiaotong University in this case-control study. All patients were newly diagnosed by clinical manifestations and confirmed to be HNSCC based on histopathological examination. The controls were selected from healthy individuals with a physical examination in the same hospital. All participants with other types of cancers and familial history of any cancers included HNSCC must be excluded. The basic characteristic of each individual was obtained from the medical records included in age, gender, lymph node metastasis status, clinical stage, BMI (body mass index), and smoking/drinking status. Each participant was told the research purpose, and informed consent was obtained from them. Our study was approved by the ethics committee of the First Affiliated Hospital of Xi'an Jiaotong University. All experiments were carried out based on the guideline of Helsinki's declaration.

### 2.2. SNP Selection and Genotyping

In our study, six SNPs (rs75267932, rs7336610, rs72640334, rs17735387, rs7318578, and rs1428) of the *MIR17HG* gene were selected by 1000 Genomes Project with MAF > 5% and *r*^2^ (the measure value of linkage disequilibrium (LD)) < 0.8 and for further genotyping. The genomic DNA from each peripheral blood sample was extracted by a whole-blood genomic DNA extraction kit (GoldMag, Xi'an, China). The NanoDrop 2000C spectrophotometer (Thermo Scientific, Waltham, USA) was performed to test the concentration and purity of the genomic DNA. PCR primers used for genotyping were designed by the Agena Bioscience Assay Design Suite software (V2.0, https://agenacx.com/online-tools/). We further identified the SNP genotyping via the Agena MassARRAY iPLEX version 4.0 platform, and the data was organized and analyzed by the Agena Bioscience TYPER version 4.0 software.

### 2.3. Statistical Analysis

All variables were examined for normal distributions using the Kolmogorov-Smirnov test. Comparisons of age and clinical characteristics between the cases and controls were, respectively, analyzed by the *t*-test. The difference of gender between the cases and controls was analyzed by the *χ*^2^ test. A chi-squared test was used to evaluate the Hardy-Weinberg equilibrium (HWE) of each SNP in the control group. Distributions of allele and genotype of SNPs in the cases and controls were analyzed by the *χ*^2^ test or exact test. The association between the *MIR17HG* gene and HNSCC susceptibility was detected by calculating ORs and 95% CIs under five inheritance models using logistic regression analysis. In addition, we investigated the correlation of the SNPs with HNSCC risk under subgroups such as age, gender, clinical stage, and HNSCC types. What is more, we also carried out a false-positive report probability (FPRP) analysis to further detect whether the significant findings were just chance or noteworthy observations [18]. Statistical analyses in this study were performed using the SPSS version 17.0 software. All statistical tests were two-tailed and *p* value <0.05 indicates statistically significant.

## 3. Results

### 3.1. Basic Characteristics of Study Participants

The basic characteristics of all participants were summarized in Table 1. This study consisted of 537 cases (207 men and 330 women) and 533 controls (204 men and 329 women). The average ages were 46.62 ± 13.67 years in controls and 46.87 ± 15.05 years in cases. There were no significant differences in age and gender between the case and control participants (*p* = 0.782; *p* = 0.950, respectively).

### 3.2. Association Analysis between *MIR17HG* Genetic Variants and HNSCC Susceptibility

The basic information of the candidate SNPs in this study was presented in Table 2. A total of six SNPs were successfully genotyped in our study. The distributions of the genotype of all SNPs in controls were in accordance with HWE (*p* > 0.05). We then investigate the association of SNPs in the *MIR17HG* gene with the risk of HNSCC under allele, codominant, dominant, recessive, and log-additive models (Table 3). It was shown that significant associations were not observed in SNPs.

### 3.3. Correlation of SNPs with HNSCC Risk Stratified by Demographic and Clinical Characteristics

We further carried out stratification analyses by age, gender, and pathological grade. When stratified by age, we found that there is no strong significant association with the risk of HNSCC (Table 4). After stratifying by gender, our result indicated that rs7336610 (TC vs. TT, OR 1.77, 95% CI = 1.09‐2.86, and *p* = 0.021; TC-CC vs. TT, OR 1.64, 95%CI = 1.04‐2.57, and *p* = 0.030) and rs1428 (AC vs. AA, OR 1.73, 95%CI = 1.07‐2.81, and *p* = 0.025; AC-CC vs. AA, OR 1.63, 95%CI = 1.04‐2.56, and *p* = 0.035) polymorphisms are strongly associated with an increased risk of HNSCC in men (Table 4). We further evaluated the relationship of the *MIR17HG* genetic variants with pathological grade of HNSCC (Table 5). rs17735387 SNP played a crucial protective role in stage III/IV HNSCC patients (GA vs. GG, OR 0.34, 95%CI = 0.12‐0.95, and *p* = 0.040; GA-AA vs. GG, OR 0.38, 95%CI = 0.15‐0.97, and *p* = 0.042) compared with stage I/II.

We finally detected the impacts of *MIR17HG* SNPs on nasopharyngeal SCC and thyroid SCC susceptibilities (Table 6). No significant associations were found between the SNPs and nasopharyngeal SCC and thyroid SCC susceptibilities.

### 3.4. FPRP Analysis

FPRP and statistical power were calculated for the positive findings for the samples. As was shown in Table 7, the association of the *MIR17HG* rs17735387 polymorphism (GA vs. GG) with the risk of stage III/IV HNSCC remained noteworthy (FPRP = 0.192), while the association of rs17735387 (GA-AA vs. GG) was not noteworthy at the prior probability level of 0.25 and FPRP threshold of 0.2 (FPRP = 0.205). Moreover, the associations of rs7336610 polymorphisms (TC vs. TT and TC-CC vs. TT) and rs1248 (CA vs. AA and AC-CC vs. AA) with HNSCC susceptibility in men were also positive at the prior probability level of 0.25 and FPRP threshold of 0.2 (FPRP = 0.106, FPRP = 0.156, FPRP = 0.138, and FPRP = 0.169, respectively).

## 4. Discussion

In this study, we assessed the association of the *MIR17HG* genetic variants (rs75267932, rs7318578, rs72640334, rs17735387, rs7336610, and rs1428) with HNSCC risk in a Chinese population. We observed that *MIR17HG* SNPs are strongly associated with HNSCC susceptibility, especially rs7336610, rs17735387, and rs1428. To our knowledge, our study is the first to investigate the correlation between *MIR17HG* variants and HNSCC risk, which suggests that *MIR17HG* genetic variants have a potential role in HNSCC progression.

**Figure 1:**
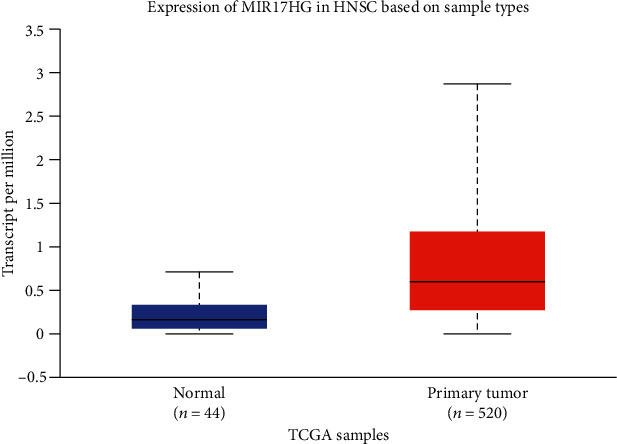
The expression of the *MIR17HG* gene between HNSCC and normal tissues from the UALCAN database. HNSCC: head and neck squamous cell carcinoma.

*MIR17HG* is a member of lncRNAs located in a region of human chromosome 13q31, which was shown to play an important role in the development and progression of several human cancers through regulating tumor growth and apoptosis [19–21]. Jiang et al. showed that the higher expression level of the *MIR17HG* gene can inhibit the growth and metastasis of colon tumors [22]. The overexpression of *MIR17HG* resulted in the evasion of apoptosis in Burkitt lymphoma cells [23]. Another study found that overexpression of *MIR17HG* was involved in a negatively regulating proapoptotic gene in the occurrence of lung cancer [21]. *MIR17HG* could affect the abnormal expression of the miR-17-92 gene. The silencing of miR-17 has a crucial role in laryngeal squamous cell carcinoma progression. *MIR17HG* plays a distinct role in HPV-related HNSCC [15]. In addition, we observed that the expression level of the *MIR17HG* gene in tumors is much higher than in normal tissues based on the UALCAN database (http://ualcan.path.uab.edu/cgibin/TCGAExResultNew2.pl?genenam=MIR17HG&ctype=HNSC) (Figure 1) [24]. We guess that the abnormal expression of MIR17HG also plays a vital role in the progression of HNSCC. As we all know, SNPs can affect the expression of genes. Thus, the study of the association between *MIR17HG* SNPs and HNSCC may help to understand whether they have a potential molecular role in the development of HNSCC.

rs7336610 and rs1428 have been identified in the correlation with human cancers at previous researches. The study of Chen et al. showed that there is a strongly increased association between rs7336610 and rs1428 and colorectal cancer susceptibility in the Chinese population in men [16]. Our study also exhibited the same association in HNSCC risk. However, Chacon-Cortes et al. found that rs7336610 is related to breast cancer susceptibility in females of Northern European [17]. In addition, it was shown that rs17735387 SNP played a crucial protective role in stage III/IV HNSCC patients compared with stage I/II. A recent study indicated that the mutation of SNP can influence the stability of lncRNA by changing its folding structure [25]. We speculate that SNPs in the *MIR17HG* gene contribute to HNSCC progression through influencing the stability.

Our study has some limitations. First, we have not detected the association of SNPs with HNSCC stratified by smoking and drinking status due to the very limited information from the medical records of participants. Next, we will collect more basic characteristics to study the associations. Second, whether the polymorphisms in the *MIR17HG* gene involved in the progression of HNSCC through affecting its functions, which is needed to explore in the subsequent work. Despite the limitations, our study supplied some scientific evidence for finding a new biomarker in the diagnosis and management of HNSCC.

## 5. Conclusions

In summary, our study showed that there is a strong association between *MIR17HG* genetic variants and HNSCC susceptibility in a Chinese population, which will provide available information for the molecular mechanism of HNSCC in the Chinese population.

## Figures and Tables

**Table 1:** Characteristic of HNSCC and healthy controls.

Characteristics	Cases (*n* = 537)	Controls (*n* = 533)	*p*
Age, years (mean ± SD)^a^	46.87 ± 15.05	46.62 ± 13.67	0.782
>46	299 (56.0%)	282 (53.0%)	
≤46	238 (44.0%)	251 (47.0%)	
Gender^b^			0.950
Male	207 (39.0%)	204 (38.0%)	
Female	330 (61.0%)	329 (62.0%)	
LN metastasis			
Node positive	103 (19.0%)		
Node negative	82 (15.0%)		
Missing	352 (66%)		
Clinical stage			
III/IV	38 (7%)		
I/II	140 (26%)		
Missing	359 (67%)		
Nasopharyngeal carcinoma	77 (14%)		
Thyroid cancer	398 (74%)		
Laryngeal carcinoma	43 (8%)		
Parotid gland carcinoma	19 (4%)		
BMI, kg/m^2^ (mean ± SD)^a^			
≤24	12 (6%)	247 (46%)	
>24	1 (0.2%)	158 (30%)	
Missing	515 (93.8%)	128 (24%)	
Smoking	90 (17%)	365 (69%)	
Drinking	46 (9%)	344 (65%)	

^a^Student's *t*-test is used. ^b^Pearson's *χ*^2^ test is used. *p* < 0.05 indicates statistical significance. HNSCC: head and neck squamous cell carcinoma; LN: lymph node; BMI: body mass index.

**Table 2:** The distribution of allele frequencies of *MIR17HG* SNPs in case and control.

SNP ID	Chromosome position	Function	Alleles (minor/major)	MAF		O (HET)	E (HET)	*p* ^a^ HWE	OR (95% CI)	*p* ^b^
				Case	Control					
rs75267932	chr13: 91351812	Exon	G/A	0.104	0.115	0.205	0.204	1.000	0.89 (0.68-1.17)	0.412
rs72640334	chr13: 91352674	Intron	A/C	0.100	0.086	0.164	0.156	0.411	1.19 (0.89-1.60)	0.238
rs7336610	chr13: 91352883	Intron	C/T	0.493	0.489	0.485	0.500	0.489	1.02 (0.86-1.21)	0.826
rs7318578	chr13: 91353215	Intron	C/A	0.289	0.277	0.400	0.401	1.000	1.06 (0.88-1.28)	0.559
rs17735387	chr13: 91353800	Intron	A/G	0.201	0.197	0.311	0.316	0.683	1.03 (0.83-1.27)	0.811
rs1428	chr13: 91354516	Exon	C/A	0.490	0.488	0.484	0.500	0.488	1.01 (0.85-1.19)	0.929

HNSCC: head and neck squamous cell carcinoma; SNP: single nucleotide polymorphisms; MAF: minor allele frequency; HWE: Hardy-Weinberg equilibrium. *p*^a^ values were calculated by exact test, and *p*^a^ < 0.05 are excluded; *p*^b^ values were calculated by two-sided *χ*^2^, and *p*^b^ < 0.05 indicates statistical significance.

**Table 3:** Association of *MIR17HG polymorphism* with HNSCC risk.

SNP ID	Model	Allele/genotype	Case *n*	Control *n*	With adjusted	
					OR (95% CI)	*p*
rs75267932						
	Allele	A	962	943	1	
		G	112	123	0.89 (0.68-1.17)	0.412
	Codominant	AA	433	417	1	
		GA	96	109	0.85 (0.63-1.15)	0.297
		GG	8	7	1.10 (0.39-3.06)	0.859
	Dominant	AA	433	417	1	
		AG-GG	104	116	0.86 (0.64-1.16)	0.338
	Recessive	AA-AG	529	526	1	
		GG	8	7	1.13 (0.41-3.15)	0.812
	Log-additive	–	–	–	0.90 (0.68-1.17)	0.421
rs72640334						
	Allele	C	959	973	**1**	
		A	107	91	1.19 (0.89-1.60)	0.238
	Codominant	CC	432	443	1	
		CA	95	87	1.12 (0.81-1.54)	0.490
		AA	6	2	3.09 (0.62-15.44)	0.169
	Dominant	CC	432	443	1	
		CA-AA	101	89	1.16 (0.85-1.59)	0.344
	Recessive	CC-CA	527	530	1	
		AA	0	2	3.04 (0.61-15.14)	0.175
	Log-additive	–	–	–	1.20 (0.89-1.61)	0.235
rs7336610						
	Allele	T	544	544	1	
		C	530	520	1.02 (0.86-1.21)	0.826
	Codominant	TT	133	143	1	
		TC	278	258	1.16 (0.87-1.55)	0.316
		CC	126	131	1.03 (0.73-1.45)	0.856
	Dominant	TT	133	143	1	
		TC-CC	404	389	1.12 (0.85-1.47)	0.429
	Recessive	TT-TC	411	401	1	
		CC	126	131	0.94 (0.71-1.24)	0.644
	Log-additive	–	–	–	1.02 (0.86-1.21)	0.833
rs7318578						
	Allele	A	764	769	1	
		C	310	295	1.06 (0.88-1.28)	0.559
	Codominant	AA	267	278	1	
		AC	230	213	1.12 (0.87-1.45)	0.360
		CC	40	41	1.02 (0.64-1.62)	0.945
	Dominant	AA	267	278	1	
		AC-CC	270	254	1.11 (0.87-1.41)	0.407
	Recessive	AA-AC	497	491	1	
		CC	40	41	0.96 (0.61-1.52)	0.875
	Log-additive	–	–	–	1.03 (0.88-1.28)	0.554
rs17735387						
	Allele	G	858	856	1	
		A	216	210	1.03 (0.83-1.27)	0.811
	Codominant	GG	344	345	1	
		GA	170	166	1.03 (0.79-1.33)	0.842
		AA	23	22	1.04 (0.57-1.91)	0.893
	Dominant	GG	344	345	1	
		AG-AA	193	188	1.03 (0.80-1.32)	0.825
	Recessive	GG-AG	514	511	1	
		AA	23	22	1.03 (0.57-1.88)	0.914
	Log-additive	–	–	–	1.02 (0.83-1.27)	0.822
rs1428						
	Allele	A	547	546	1	
		C	525	520	1.01 (0.85-1.19)	0.929
	Codominant	AA	137	144	1	
		AC	273	258	1.11 (0.83-1.49)	0.464
		CC	126	131	1.01 (0.72-1.42)	0.957
	Dominant	AA	137	144	1	
		AC-CC	399	389	1.08 (0.82-1.42)	0.586
	Recessive	AA-AC	410	402	1	
		CC	126	131	0.94 (0.71-1.25)	0.670
	Log-additive	–	–	–	1.01 (0.85-1.19)	0.935

HNSCC: head and neck squamous cell carcinoma; CI: confidence interval; OR: odds ratio; SNP: single nucleotide polymorphism. *p* values were calculated by unconditional logistic regression analysis with adjustment for age and gender. *p* < 0.05 indicates statistical significance. Highlighted in bold indicates the significant association between SNPs and HNSCC risk.

**Table 4:** The relationship of *MIR17HG* variants with HNSCC stratified by age and gender.

SNP ID	Allele/genotype	>46 years		≤46 years		Men		Women	
		OR (95% CI)	*p*	OR (95% CI)	*p*	OR (95% CI)	*p*	OR (95% CI)	*p*
rs75267932	A	1		1		1		1	
	G	1.05 (0.72-1.53)	0.810	0.76 (0.51-1.12)	0.166	0.93 (0.60-1.46)	0.765	0.87 (0.62-1.22)	0.422
	AA	1		1		1		1	
	GA	0.98 (0.64-1.50)	0.937	0.71 (0.45-1.10)	0.125	1.24 (0.75-2.04)	0.407	0.68 (0.46-1.00)	0.050
	GG	1.52 (0.36-6.47)	0.570	0.81 (0.18-3.71)	0.790	/	/	3.77 (0.79-17.98)	0.096
	AG-GG	1.01 (0.67-1.53)	0.950	0.71 (0.46-1.10)	0.125	1.08 (0.66-1.75)	0.761	0.76 (0.52-1.11)	0.149
rs72640334	C	1		1		1		1	
	A	1.07 (0.72-1.59)	0.724	1.36 (0.87-2.11)	0.174	0.91 (0.57-1.46)	0.705	1.43 (0.97-2.09)	0.068
	CC	1		1		1		1	
	CA	1.14 (0.75-1.75)	0.536	1.06 (0.65-1.73)	0.814	0.96 (0.58-1.57)	0.858	1.25 (0.82-1.90)	0.295
	AA	0.47 (0.04-5.23)	0.539	/	/	/	/	6.37 (0.76-53.39)	0.088
	CA-AA	1.12 (0.73-1.70)	0.608	1.20 (0.75-1.93)	0.450	0.93 (0.57-1.53)	0.780	1.35 (0.90-2.04)	0.147
rs7336610	T	1		1		1		1	
	C	1.07 (0.85-1.35)	0.559	1.13 (0.88-1.45)	0.352	1.19 (0.91-1.57)	0.210	1.15 (0.93-1.43)	0.204
	TT	1		1		1		1	
	TC	1.41 (0.95-2.10)	0.091	1.14 (0.74-1.76)	0.564	**1.77 (1.09-2.86)**	**0.021**	1.12 (0.77-1.62)	0.550
	CC	1.14 (0.73-1.78)	0.568	1.33 (0.78-2.26)	0.295	1.43 (0.83-2.46)	0.203	1.33 (0.86-2.07)	0.199
	TC-CC	1.30 (0.90-1.89)	0.161	1.19 (0.78-1.80)	0.419	**1.64 (1.04-2.57)**	**0.032**	1.18 (0.83-1.68)	0.348
rs7318578	A	1		1		1		1	
	C	1.05 (0.81-1.35)	0.711	1.07 (0.81-1.41)	0.655	0.93 (0.69-1.25)	0.626	1.15 (0.90-1.46)	0.256
	AA	1		1		1		1	
	CA	1.11 (0.79-1.56)	0.561	1.14 (0.79-1.66)	0.487	0.94 (0.63-1.42)	0.776	1.25 (0.91-1.73)	0.166
	CC	1.02 (0.54-1.94)	0.944	0.95 (0.48-1.90)	0.894	0.84 (0.40-1.73)	0.634	1.16 (0.63-2.13)	0.642
	AC-CC	1.09 (0.79-1.52)	0.591	1.11 (0.78-1.59)	0.568	0.92 (0.63-1.36)	0.692	1.24 (0.91-1.68)	0.171
rs17735387	G	1		1		1		1	
	A	0.98 (0.74-1.31)	0.894	1.08 (0.79-1.48)	0.622	0.92 (0.65-1.30)	0.649	1.10 (0.84-1.43)	0.510
	GG	1		1		1		1	
	GA	0.94 (0.66-1.34)	0.740	1.17 (0.80-1.73)	0.422	1.00 (0.65-1.53)	0.999	1.04 (0.75-1.45)	0.799
	AA	1.04 (0.47-2.33)	0.916	1.09 (0.43-2.78)	0.857	0.68 (0.25-1.84)	0.446	1.36 (0.62-2.96)	0.440
	GA-AA	0.95 (0.68-1.34)	0.785	1.16 (0.80-1.69)	0.428	0.96 (0.64-1.44)	0.827	1.08 (0.78-1.48)	0.651
rs1428	A	1		1		1		1	
	C	1.08 (0.86-1.36)	0.523	1.11 (0.86-1.42)	0.424	1.20 (0.92-1.58)	0.185	1.14 (0.92-1.41)	0.246
	AA	1		1		1		1	
	CA	1.38 (0.93-2.06)	0.112	1.07 (0.70-1.65)	0.750	**1.73 (1.07-2.81)**	**0.025**	1.07 (0.74-1.55)	0.713
	CC	1.15 (0.74-1.80)	0.533	1.28 (0.75-2.16)	0.367	1.45 (0.84-2.50)	0.180	1.30 (0.84-2.01)	0.239
	AC-CC	1.29 (0.89-1.87)	0.177	1.13 (0.75-1.70)	0.573	**1.63 (1.04-2.56)**	**0.035**	1.14 (0.81-1.61)	0.463

HNSCC: head and neck squamous cell carcinoma. *p* values were calculated by unconditional logistic regression analysis with adjustment for age and gender. *p* < 0.05 indicates statistical significance. Highlighted in bold indicates the significant association between SNPs and HNSCC risk.

**Table 5:** The relationship of *MIR17HG* polymorphisms with HNSCC stratified by pathological grade.

SNP ID	Allele/genotype	III-IV (*n*)	I-II (*n*)	OR (95% CI)	*p*
rs75267932	A	70	253	1	
	G	6	27	0.80 (0.32-2.02)	0.641
	AA	32	113	1	
	GA	6	27	/	/
	GG	0	0	/	/
	AG-GG	6	27	0.65 (0.23-1.86)	0.420

rs72640334	C	66	251	1	
	A	10	29	1.31 (0.61-2.83)	0.488
	CC	28	112	1	
	CA	10	27	1.90 (0.76-4.71)	0.169
	AA	0	1	/	/
	CA-AA	10	28	1.84 (0.74-4.55)	0.189

rs7336610	T	33	144	1	
	C	43	136	1.38 (0.83-2.30)	0.216
	TT	8	36	1	
	TC	17	72	0.98 (0.37-2.60)	0.963
	CC	13	32	1.67 (0.58-4.81)	0.342
	TC-CC	30	104	1.19 (0.48-2.97)	0.706

rs7318578	A	53	195	1	
	C	23	85	1.00 (0.57-1.73)	0.987
	AA	20	65	1	
	CA	13	65	0.84 (0.36-1.93)	0.676
	CC	5	10	2.25 (0.62-8.16)	0.218
	AC-CC	18	75	1.01 (0.47-2.20)	0.973

rs17735387	G	66	223	1	
	A	10	57	0.59 (0.29-1.23)	0.154
	GG	30	92	1	
	GA	6	39	**0.34 (0.12-0.95)**	**0.040**
	AA	2	9	0.60 (0.11-3.16)	0.549
	GA-AA	8	48	**0.38 (0.15-0.97)**	**0.042**

rs1428	A	33	143	1	
	C	43	135	1.38 (0.83-2.30)	0.215
	AA	8	36	1	
	CA	17	71	0.99 (0.37-2.64)	0.980
	CC	13	32	1.69 (0.59-4.87)	0.330
	AC-CC	30	103	1.21 (0.48-3.02)	0.684

*p* values were calculated by unconditional logistic regression analysis with adjustment for age and gender. *p* < 0.05 indicates statistical significance. Highlighted in bold indicates the significant association between SNPs and HNSCC risk.

**Table 6:** The relationship of *MIR17HG* variants with nasopharyngeal SCC and thyroid SCC risk.

SNP ID	Allele/genotype	Nasopharyngeal SCC				Thyroid SCC			
		Case	Control	OR (95% CI)	*p*	Case	Control	OR (95% CI)	*p*
rs75267932	A	135	943	1		714	943	1	
	G	19	123	1.08 (0.64-1.81)	0.773	82	123	0.88 (0.66-1.18)	0.399
	AA	59	417	1		323	417	1	
	GA	17	109	1.24 (0.68-2.24)	0.485	68	109	0.79 (0.56-1.11)	0.173
	GG	1	7	0.87 (0.10-7.39)	0.898	7	7	1.37 (0.47-3.98)	0.562
	AG-GG	18	116	1.21 (0.68-2.16)	0.524	75	116	0.82 (0.59-1.14)	0.247
rs72640334	C	132	973	1		713	973	1	
	A	18	91	1.46 (0.85-2.50)	0.167	81	91	1.22 (0.89-1.66)	0.225
	CC	58	443	1		321	443	1	
	CA	16	87	1.29 (0.70-2.37)	0.419	71	87	1.15 (0.81-1.63)	0.435
	AA	1	2	3.65 (0.29-46.40)	0.318	5	2	3.09 (0.58-16.38)	0.184
	CA-AA	17	89	1.34 (0.73-2.43)	0.342	76	89	1.20 (0.85-1.68)	0.306
rs7336610	T	70	544	1		408	544	1	
	C	84	520	1.26 (0.89-1.76)	0.188	388	520	0.99 (0.83-1.20)	0.956
	TT	14	143	1		102	143	1	
	TC	42	258	1.72 (0.90-3.29)	0.102	204	258	1.09 (0.79-1.50)	0.594
	CC	21	131	1.45 (0.70-3.01)	0.319	92	131	1.00 (0.69-1.46)	0.986
	TC-CC	63	389	1.62 (0.87-3.01)	0.127	296	389	1.06 (0.79-1.43)	0.697
rs7318578	A	102	769	1		573	769	1	
	C	52	295	1.33 (0.93-1.91)	0.121	223	295	1.02 (0.83-1.25)	0.890
	AA	32	278	1		202	278	1	
	CA	38	213	1.46 (0.88-2.44)	0.147	169	213	1.10 (0.84-1.45)	0.494
	CC	7	41	1.36 (0.55-3.35)	0.503	27	41	0.89 (0.53-1.50)	0.664
	AC-CC	45	254	1.45 (0.88-2.37)	0.144	196	254	1.07 (0.82-1.39)	0.632
rs17735387	G	122	856	1		634	856	1	
	A	32	210	1.07 (0.70-1.62)	0.754	162	210	1.04 (0.83-1.31)	0.728
	GG	48	345	1		253	345	1	
	GA	26	166	1.14 (0.68-1.93)	0.621	128	166	1.06 (0.79-1.40)	0.708
	AA	3	22	0.88 (0.25-3.10)	0.843	17	22	1.11 (0.57-2.14)	0.762
	GA-AA	29	188	1.11 (0.67-1.83)	0.692	145	188	1.06 (0.81-1.40)	0.667
rs1428	A	70	546	1		412	546	1	
	C	84	520	1.26 (0.90-1.77)	0.181	384	520	0.98 (0.81-1.18)	0.818
	AA	14	144	1		106	144	1	
	CA	42	258	1.73 (0.90-3.31)	0.099	200	258	1.03 (0.75-1.42)	0.838
	CC	21	131	1.46 (0.70-3.02)	0.314	92	131	0.97 (0.67-1.41)	0.878
	AC-CC	63	389	1.63 (0.88-3.02)	0.123	292	389	1.01 (0.75-1.36)	0.933

Nasopharyngeal SCC: nasopharyngeal squamous cell carcinoma; thyroid SCC: thyroid squamous cell carcinoma. *p* values were calculated by unconditional logistic regression analysis with adjustment for age and gender. *p* < 0.05 indicates statistical significance. Highlighted in bold indicates the significant association between SNPs and HNSCC risk.

**Table 7:** False-positive report probability analysis for the significant findings between *MIR17HG* variants and HNSCC risk.

Genotype and variables	OR (95% CI)	*p* value^a^	Statistical power^b^	Prior probability				
				0.25	0.1	0.01	0.001	0.0001
III-IV								
rs17735387 G>A								
GA vs. GG	0.34 (0.12-0.95)	0.040	0.308	0.192^c^	0.416	0.887	0.988	0.999
GA-AA vs. GG	0.38 (0.15-0.97)	0.042	0.329	0.205	0.436	0.895	0.988	0.999
Men								
rs7336610 T>C								
TC vs. TT	1.77 (1.09-2.86)	0.021	0.487	0.106^c^	0.262	0.796	0.975	0.997
TC-CC vs. TT	1.64 (1.04-2.57)	0.032	0.031	0.156^c^	0.357	0.859	0.984	0.998
rs1428 A>C								
CA vs. AA	1.73 (1.07-2.81)	0.025	0.410	0.138^c^	0.325	0.841	0.982	0.998
AC-CC vs. AA	1.63 (1.04-2.56)	0.035	0.034	0.169^c^	0.379	0.870	0.985	0.999

HNSCC: head and neck squamous cell carcinoma. *p* value^a^ was calculated by unconditional logistic regression analysis with adjustment for age and gender. Statistical power^b^ was calculated using the number of observations in the subgroup and the OR and *p* values in this table. ^c^The level of false-positive report probability threshold was set at 0.2, and noteworthy findings are presented.

## Data Availability

Participant informed consent statements did not seek consent for data to be made publicly available; however, data will be made available to individual researchers upon reasonable request.
